# Quaternized Chitosan-Capped Mesoporous Silica Nanoparticles as Nanocarriers for Controlled Pesticide Release

**DOI:** 10.3390/nano6070126

**Published:** 2016-06-28

**Authors:** Lidong Cao, Huirong Zhang, Chong Cao, Jiakun Zhang, Fengmin Li, Qiliang Huang

**Affiliations:** Key Laboratory of Integrated Pest Management in Crops, Ministry of Agriculture, Institute of Plant Protection, Chinese Academy of Agricultural Sciences, No. 2 Yuanmingyuan West Road, Beijing 100193, China; caolidong@caas.cn (L.C.); 1988zhanghuirong@163.com (H.Z.); ccao@ippcaas.cn (C.C.); Zhangjiakun0521@126.com (J.Z.); fmli@ippcaas.cn (F.L.)

**Keywords:** pesticide, pyraclostrobin, mesoporous silica nanoparticles, HTCC, release, fungicidal activity

## Abstract

Nanotechnology-based pesticide formulations would ensure effective utilization of agricultural inputs. In the present work, mesoporous silica nanoparticles (MSNs) with particle diameters of ~110 nm and pore sizes of ~3.7 nm were synthesized via a liquid crystal templating mechanism. A water-soluble chitosan (CS) derivative (*N*-(2-hydroxyl)propyl-3-trimethyl ammonium CS chloride, HTCC) was successfully capped on the surface of pyraclostrobin-loaded MSNs. The physicochemical and structural analyses showed that the electrostatic interactions and hydrogen bonding were the major forces responsible for the formation of HTCC-capped MSNs. HTCC coating greatly improved the loading efficiency (LC) (to 40.3%) compared to using bare MSNs as a single encapsulant (26.7%). The microstructure of the nanoparticles was revealed by scanning electron microscopy (SEM) and transmission electron microscopy (TEM). The pyraclostrobin-loaded nanoparticles showed an initial burst and subsequent sustained release behavior. HTCC-capped MSNs released faster than bare MSNs in the initial stage. Pyraclostrobin-loaded HTCC-capped MSNs with half doses of pyraclostrobin technical demonstrated almost the same fungicidal activity against *Phomopsis asparagi* (Sacc.), which obviously reduced the applied pesticide and enhanced the utilization efficiency. Therefore, HTCC-decorated MSNs demonstrated great potential as nanocarriers in agrochemical applications.

## 1. Introduction

Pesticides, widely used to kill insects, weeds, fungi and other organisms, bring great benefits to society [1]. However, depending on the environmental conditions and mode of application, more than 90% of the applied pesticides are either lost in the environment or unable to reach the target area required for pest control, which not only increases the cost but also leads to environmental pollution [2]. Therefore, improved utilization of pesticides has been a very active field of research over the past decade. Inspired by the achievement of smart drug delivery systems, controlled-release formulations of pesticides are a pertinent alternative for delivering the active ingredient slowly and continuously for longer durations to a specified target at a desired rate [3,4,5]. In the process of technology advancement, nano-delivery systems, which can provide better penetration through cuticles and allow precise pesticide targeting, seem promising and have great potential as delivery options for the current challenges that modern pesticides face. In recent years, the development of nanocarriers for the targeted delivery and controlled release of agrochemicals has gained considerable attention [6,7,8,9,10,11].

Recently, we have developed several pesticide nanoparticles that use amphiphilic chitosan (CS)-poly(lactide) and CS-poly(lactide)-1,2-dipalmitoylsn-glycero-3-phosphoethanolamine copolymers as carriers to improve stability and sustained release performance [12,13,14]. However, the loading content (LC) of pesticides was not satisfactory when only organic polymers were employed (less than 20%). Hybridization with inorganic materials with large surface areas can provide a suitable solution for this limitation. Since the discovery of Mobil Crystalline Material 41 (MCM-41), research on and development of mesoporous silica nanoparticles (MSNs) has gained worldwide interest due to MSNs’ unique properties, such as biocompatibility, low cost, large surface area, tunable pore size for high loading capacity, and ability for targeted and controlled release with surface functionalization and polymer coatings [15,16,17,18,19].

Several studies have recently described the controlled release of pesticides using silica-based materials. Wen and co-workers employed porous hollow silica nanoparticles as pesticide carriers to study the controlled release behavior of avermectin [20,21]. Chen reported a slow-release formula of a potential biological pesticide, pyoluteorin, prepared using mesoporous nanoparticles as carriers to avoid its fast degradation [22]. The insecticide imidacloprid was effectively loaded into MSNs with different pore sizes, morphologies and mesoporous structures for termite control [23]. Bernardos reported that essential oil components loaded into MSNs had sustained antifungal activities against *Aspergillus niger* [24]. In the case of unmodified MSNs, which have only silanol groups on the pore channel walls, pesticide molecules that had diffused into the MSNs’ nanopores could only release in a slow and sustained mannner. For controlled release in response to external stimuli and other functionalities, some responsive molecules can be modified on the MSNs’ surface or nanopores [25]. Prado reported nanosized silica modified with carboxylic acid as a support for the controlled release of the herbicides 2,4-D and picloram [26]. Zhang recently developed a novel functionalized microcapsule using silica cross-linked with alginate, and some beneficial elements to crops. The prepared microcapsules that had a high loading efficiency for prochloraz (about 30% *w*/*w*) could effectively protect prochloraz against degradation under UV irradiation and alkaline conditions, and showed sustainable release for at least 60 days [27]. Yi established functionalized MSNs with redox-responsive, short-chain decanethiol gatekeepers for agrochemical salicylic acid (SA) delivery. The release rate of SA under a certain amount of glutathione (GSH) was obviously higher than that without GSH [28].

**Figure 1:**
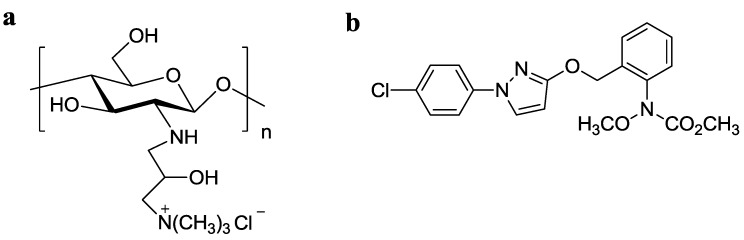
Molecular structures of *N*-2-hydroxypropyl trimethyl ammonium chloride chitosan (HTCC) (**a**) and pyraclostrobin (**b**).

Natural polymers are good shell materials for microcapsules due to their high biocompatibility, low toxicity and versatile chemical and physical properties. CS, a biopolymer obtained through the deacetylation of chitin, has distinctive biological properties and had been widely used in sustainable agriculture [29]. Among the derivates of CS, quaternized CS, especially *N*-(2-Hydroxyl)propyl-3-trimethylammonium CS chloride (HTCC) (Figure 1a), has attracted much attention because of its properties, including cationic charge retention at neutral pH, good water solubility, antibacterial activity, mucoadhesivity and permeability-enhancing properties [30]. Therefore, coating of HTCC with positive charge onto negatively surface-charged MSNs through electrostatic interaction without complex chemical grafting would be an ideal strategy for the surface modification of MSNs as pesticide carriers. Pyraclostrobin (Figure 1b) (Py), which is a broad-spectrum, high-efficiency and low-toxicity novel strobilurin fungicide, was selected as a model pesticide to explore the feasibility of HTCC capped-MSNs as controlled release carriers.

In the present work, we constructed HTCC-capped MSNs loaded with poorly water-soluble pyraclostrobin through facile self-assembly. Electrostatic interaction of positively-charged HTCC with oppositely-charged MSNs is the primary driving force for nanoparticle formation. Subsequently, the nanoparticles size, surface morphology and pesticide loading content were investigated. The pesticide release properties under aqueous solutions were also studied in vitro. Moreover, the fungicidal activity of pyraclostrobin-loaded nanoparticles against the fungus *Phomopsis asparagi* (Sacc.) was also explored.

## 2. Results and Discussion

### 2.1. Preparation and Characterization of Pyraclostrobin-Loaded HTCC-Capped MSNs

**Figure 2:**
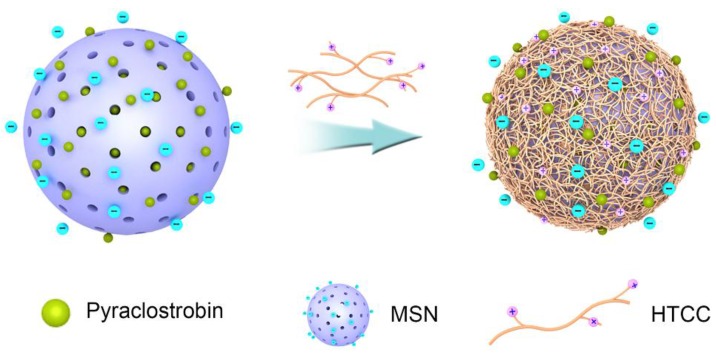
Schematic illustration of the pyraclostrobin-loaded HTCC-capped mesoporous silica nanoparticles (MSNs) (Py@MSNs-HTCC) formation process.

Figure 2 shows the schematic illustration of the pyraclostrobin-loaded HTCC-capped MSN construction process. In the present study, MSNs were synthesized by the liquid crystal templating mechanism using cetyltrimethylammonium bromide (CTAB) as the surfactant and tetraethyl orthosilicate (TEOS) as the silica precursor in basic conditions. MSNs were loaded with pesticide molecules by simple immersion in a concentrated methanol solution of pyraclostrobin. In the aqueous solution, the MSNs showed negative charges, verified by the zeta potential of −18.2 mV that was determined by ZetaSizer Nano ZS Analyzer (Malvern Instruments Ltd, Malvern, England), and HTCC was positively charged. HTCC was successfully wrapped onto the MSN surface by the driving forces of electrostatic and hydrogen bonding interactions.

#### 2.1.1. Optimization of Pesticide Loading

High pesticide loading contents in sustained and controlled formulations are important for practical applications in plant protection. When bare MSNs without surface functionalization were used for biocide loading, the uptake and release behaviors of the guest molecules mainly depended on the pore size, surface area and mesoporous structure of MSNs [23]. In the present study, HTCC coating influenced pesticide loading. The optimization results of pyraclostrobin loading are summarized in Table 1. When the amounts of MSNs and HTCC were fixed, the loading content was increased with the higher mass ratio of pyraclostrobin to MSNs (entries 1−4 and 5−6, Table 1). The loading content was only 26.7% without HTCC, which demonstrated that HTCC coating can prominently increase pesticide loading (entry 8, Table 1). It was supposed that the pesticide molecule in solution outside the MSNs could also be entrapped in the HTCC layer during the coating process onto the MSN surface. The effect of the HTCC amount on the loading content was also investigated. Additional HTCC did not obviously enhance LC, which implied a saturation coating of HTCC when the MSN amount was constant. This hypothesis could be partially proved by the basically constant zeta potential of the pesticide-loaded nanoparticles. The positive zeta potential and negative counterpart without decoration, on the other hand, affirmed the successful coating of HTCC. Based on the optimization, an equivalent mass ratio of MSNs, pyraclostrobin and HTCC was selected for preparation and characterization.

#### 2.1.2. Characterization

**Figure 3:**
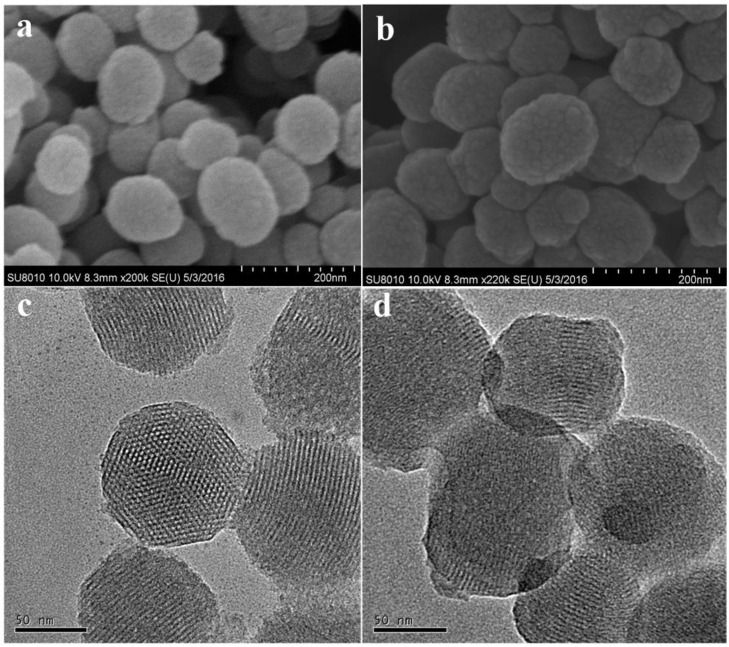
Scanning electron microscopy (SEM) images of MSNs (**a**) and pyraclostrobin-loaded HTCC-capped MSNs (**b**); Transmission electron microscopy (TEM) images of MSNs (**c**) and pyraclostrobin-loaded HTCC-capped MSNs (**d**).

The morphology of the MSNs and Py@MSNs-HTCC was observed using scanning electron microscopy (SEM) (SU8000, Hitachi, Ltd., Tokyo, Japan) and transmission electron microscopy (TEM) (JEM-200CX, Jeol Ltd., Tokyo, Japan). SEM micrographs showed that as-synthesized MSNs were nearly monodispersed spherical nanoparticles with relatively smooth surfaces (Figure 3a). The surfaces are, obviously, rough after pesticide loading and HTCC coating (Figure 3b); however, the ability of SEM to investigate the nanoparticles surface structure in detail is limited. To verify the mesoporous structure of MSNs, TEM was also used. Highly ordered mesoporous structures with hexagonal arrays of the MSNs are clearly shown, which is the characteristic of MCM-41-type MSNs (Figure 3c). Coating by the thin layer of HTCC around the particles can be clearly seen in Figure 3d. The rough surface and loss of mesoporous surface structure suggests successful wrapping with HTCC. Regarding the particles size, there were no big differences between the coated and uncoated particles, with the average diameters of 113 and 110 nm, respectively, determined by statistical analysis of the SEM images of more than 200 nanoparticles. The histograms of particle size distributions of MSNs and Py@MSNs-HTCC are shown in Supplementary Information (Figures S1 and S2). The diameters of nanoparticles were also measured using a ZetaSizer Nano ZS Analyzer, based on dynamic light scattering (DLS). The average diameters of MSNs and Py@MSNs-HTCC are 288 and 299 nm, respectively. These values are much higher than that determined by SEM. We thought that the diameter determined by DLS should be the hydrate of nanoparticles and the diameter of SEM should reflect the real entity.

**Figure 4:**
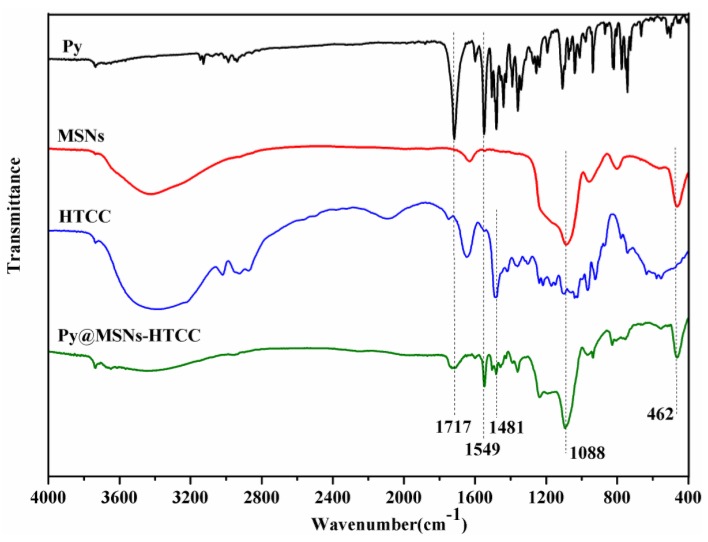
Fourier transform infrared (FTIR) spectra of pyraclostrobin (Py), MSNs, HTCC and Py@MSNs-HTCC.

The Fourier transform infrared (FTIR) spectroscopy of pyraclostrobin technical, MSNs, HTCC and the Py@MSNs-HTCC composite is shown in Figure 4. Pyraclostrobin shows strong band absorption at 1717 and 1549 cm^−1^, which corresponds to the carbonyl group and benzene ring skeleton vibration present in pyraclostrobin. The 1088 cm^−1^ broad absorption band was ascribed to Si–O–Si (siloxane) strething vibrations. Almost no C–H stretch peaks are present in the MSN samples, implying that the surfactant template of CTAB was completely extracted [31]. The peak at 1481 cm^−1^ for the HTCC spectra can be assigned to the C–H bending vibration of –N(CH_3_)_3_^+^, confirming the existence of a quaternized amine group in the CS backbone [32]. The characteristic band absorptions of pyraclostrobin, MSNs and HTCC can be found in Py@MSNs-HTCC, which confirmed the successful coating of MSNs with HTCC and the loading of pyraclostrobin.

**Figure 5:**
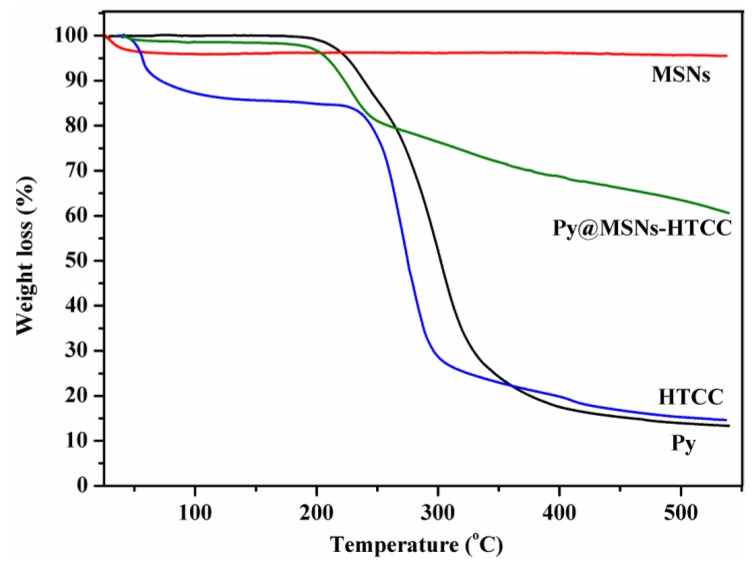
Thermogravimetric analysis (TGA) of pyraclostrobin, MSNs, HTCC and Py@MSNs-HTCC.

Thermogravimetric analysis (TGA) has frequently been used to study the decomposition pattern and thermal stability of chemicals and materials. Figure 5 displays TG analysis of pyraclostrobin technical, MSNs, HTCC and the Py@MSNs-HTCC. MSNs are thermo-stable and almost keep constant weight in the temperature range studied. Pyraclostrobin technical shows a sharp weight loss that started at about 200 °C due to the decomposition of pyraclostrobin. HTCC has two distinct weight loss stages, which started at about 50 and 240 °C. The first weight loss for HTCC is ascribed to the vaporization of bound water and residual organic solvent during the preparation of HTCC [33,34]; the second one is due to the thermal decomposition of HTCC. The two-stage weight loss in pyraclostrobin loaded HTCC-capped MSNs is noteworthy. The first stage loss at around 200 °C is mainly due to the decomposition of pyraclostrobin and the second weight loss at about 240 °C is principally attributed to decomposition of HTCC, which is further evidence that MSNs were successfully coated with HTCC and loaded with pyraclostrobin.

**Figure 6:**
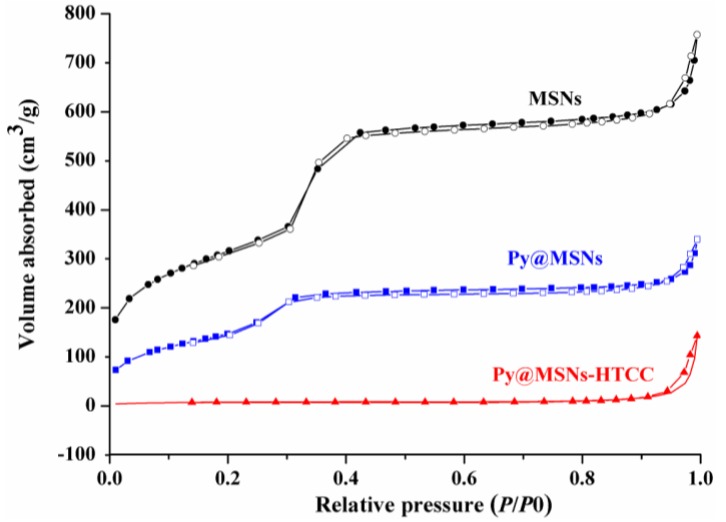
Nitrogen adsorption-desorption isotherms of MSNs, Py@MSNs and Py@MSNs-HTCC.

**Figure 7:**
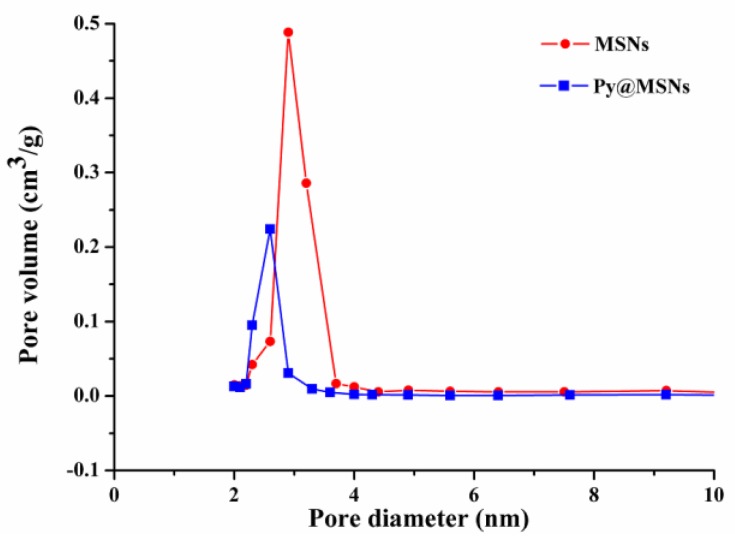
Barrett–Joyner–Halenda (BJH) pore-size-distribution curves of MSNs and Py@MSNs.

To confirm the HTCC gating potential, Brunauer–Emmett–Teller (BET) surface area analysis and Barrett–Joyner–Halenda (BJH) pore size and volume analysis were used to confirm the particles’ mesoporous structure. The values for the BET specific surface area (*S*_BET_), the total pore volume (*V*_t_) and the BJH pore diameter (*D*_BJH_) are given Table 2. Figure 6 shows the nitrogen adsorption and desorption isotherms of MSNs, Py@MSNs and Py@MSNs-HTCC. The type IV isotherm curve with an obvious step between 0.3 and 0.4 of P/P_0_, and the pore size distribution curve (Figure 7) indicate that MSNs possess a well-defined mesoporous structure. Pyraclostrobin-loaded MSNs display obvious reductions in adsorption capacity, surface area, pore volume and diameter. After HTCC coating, the amount of nitrogen adsorption further decreases due to the sealing effect of the outside HTCC layer. The surface area and pore volume were sharply reduced to 29 m^2^/g and 0.22 cm^3^/g, respectively, which provided further supportive evidence of HTCC coating. Lee has reported that the BET surface area was dramatically reduced from 1198 m^2^/g to 20 m^2^/g after heavy aminotriazole loading of copper-impregnated MSNs [31]. Additionally, the loss of the hysteresis loop affirms that the pores were blocked by the polymer coating and lost their mesoporous structure characteristics. The successful HTCC coating and pyraclostrobin loading were further verified by calcination of the Py@MSNs-HTCC nanoparticles, after which it regained its original surface area (960 m^2^/g) and pore size (3.9 nm) (Table 2, Figures S3 and S4). The ordered mesoporous structures were not disturbed after loading and calcination, which was also proved by FTIR spectra (Figure S5) and TGA (Figure S6).

### 2.2. In Vitro Release of Pyraclostrobin

**Figure 8:**
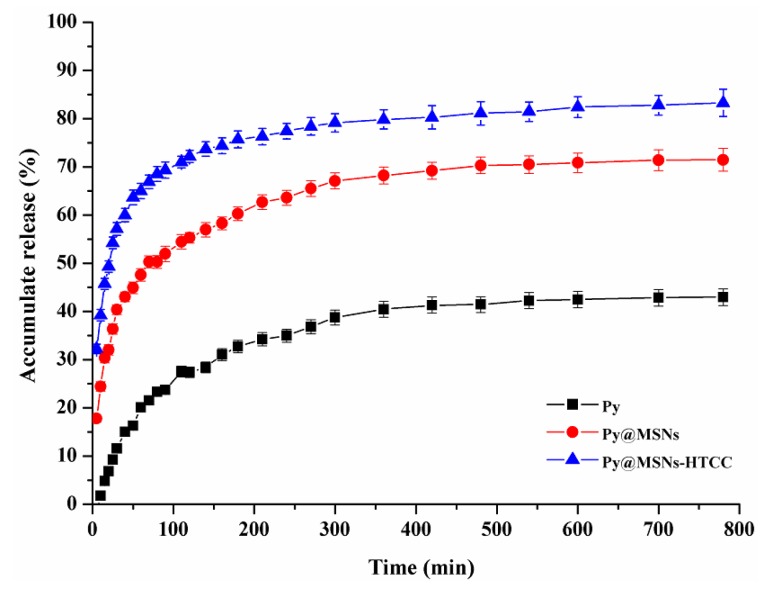
Release profiles of pyraclostrobin (Py) from pyraclostrobin technical, Py@MSNs and Py@MSNs-HTCC at room temperature. Each data point represents the mean ± standard deviation of three determinations.

The in vitro release profiles of pyraclostrobin from pyraclostrobin technical, Py@MSNs and Py@MSNs-HTCC are shown in Figure 8. Pyraclostrobin is poorly water-soluble. In the present study, 30% aqueous methanol solution plus 0.5% Tween-80 emulsifier was adopted as the release medium to explore the release behavior. The amount of pyraclostrobin technical in the release medium at the sampling time of 600 min accumulated to no more than 40%. On the contrary, pesticide loaded in MSNs and HTCC-coated MSNs was quickly released. The release improvement may be largely attributed to the mesopores of MSNs changing the crystalline state of the guest molecule to a non-crystalline state, which is known to improve the dissolution rate [35]. All the pyraclostrobin-loaded nanoparticles showed the same release profiles, specifically a high initial burst release followed by a slow, sustained release. The initial burst releases are attributed to the presence of pyraclostrobin in the external surface of the nanoparticles, which satisfies the need for immediate treatment after application. The biocompatible polymer-coated nanoparticles are of great interest for regulating the delivery and controlled release of drugs and pesticides. As shown in Figure 8, HTCC-capped MSNs released higher amounts (72%) of pyraclostrobin than bare MSNs (55%) within 120 min, this was because the pyraclostrobin molecules were encapsulated inside the pores of MSNs and also absorbed by the outside HTCC layers. In the aqueous release medium, the guest molecule on the external HTCC layers could release quickly; on the other hand, HTCC were swelled to open the pores of MSNs and probably partially dissolved to leave the MSNs, and thus faster release was observed within the short initial sampling time. The surface properties of MSNs could dramatically affect the cargo release behaviors. Lee reported a facile synthesis of MSNs with different densities of positive surface charges and the controlled release of anionic drug molecules triggered by strong electrostatic repulsion [36]. In our system, although the coating polymer is positively charged, the loaded molecular pyraclostrobin is charge neutral. The hydrogen bonding interaction between the pesticide molecules and the carrier surface might be the major parameter that regulates the release; however, the important concept and results reported by Lee can inspire us to adopt the anionic pesticide molecule to investigate the functionality of HTCC in controlling the loading content and release behavior in future research.

### 2.3. Fungicidal Activity of Pyraclostrobin-Loaded HTCC-Capped MSNs

**Figure 9:**
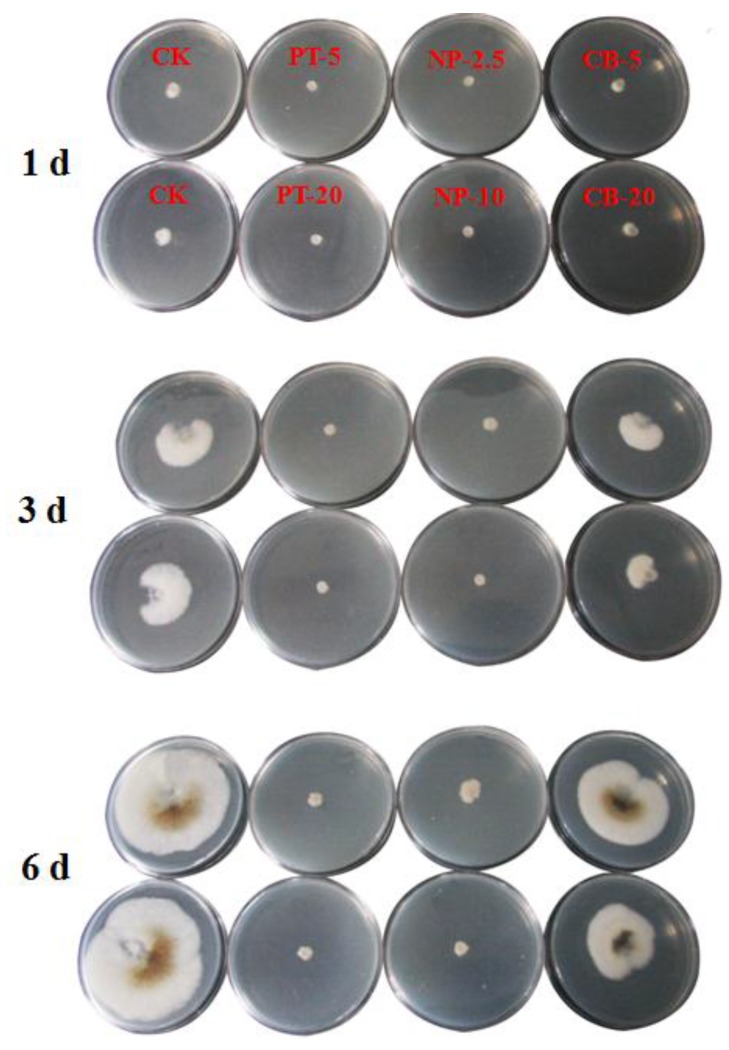
The images of the fungicidal activity of pyraclostrobin technical (PT), pyraclostrobin-loaded HTCC-capped MSNs (NP) and blank carrier MSNs-HTCC (BC) against *P*. *asparagi* on the 1st, 3rd and 6th days. The numbers after the abbreviations are the corresponding concentrations (mg/L).

In the present study, the fungicidal activity of pyraclostrobin-loaded nanoparticles against *P*. *asparagi* was determined by the growth rate method. For comparison, pyraclostrobin technical and carrier material MSNs-HTCC were also tested. The fungicidal activities of pyraclostrobin technical, Py@MSNs-HTCC and carrier blank MSNs-HTCC against *P*. *asparagi* are summarized in Table 3. The images of fungicidal activity against *P*. *asparagi* on the 1st, 3rd and 6th days are shown in Figure 9. The concentrations of the active ingredient were set to 5.0 and 20.0 mg/L for pyraclostrobin technical, and 2.5 and 10.0 mg/L for Py@MSNs-HTCC to screen for fungicidal activity. For the nanoparticles, the inhibition percentages were 87.72% and 94.74% under the concentrations of 2.5 and 10.0 mg/L, respectively, on the 6th day. Figure 9 clearly indicates that pyraclostrobin-loaded nanoparticles demonstrated almost the same activity even under half doses of pyraclostrobin technical, which obviously reduced the pesticide amounts applied and enhanced the utilization efficiency. It is worth noting that blank carrier MSN-HTCCs also had some fungicidal activity against *P*. *asparagi*, which is an interesting phenomenon that deserves further research.

## 3. Experimental Section

### 3.1. Materials

CTAB (99%) was purchased from J&K Scientific Ltd., Beijing, China. TEOS was purchased from Fluorochem Ltd., Hadfield, UK. HTCC was supplied by Tianhua Biological Agents Ltd., Dongying, China. The model pesticide, pyraclostrobin technical (97%) was supplied by BASF (Ludwigshafen) in Germany. The *P*. *asparagi* strain was provided by the Pesticide Bioassay Lab in Institute of Plant Protection, Chinese Academy of Agricultural Sciences (Beijing, China). All other chemicals and reagents were commercially available and used without further processing.

### 3.2. Characterization

FTIR analysis was recorded on a spectrophotometer (NICOLET 6700, Thermo Scientific, Waltham, MA, USA) with a potassium bromide pellet, and recorded over the spectral region of 400 to 4000 cm^−1^.

The nanoparticle zeta potential were measured using a ZetaSizer Nano ZS Analyzer (Zetasizer Nano ZS, Malvern Instruments Ltd., Malvern, UK), based on DLS.

Thermogravimetric analysis was performed with a Perkin Elmer Pyris Diamond (Woodland, CA, USA) TG/DTA from room temperature to 550 °C at a heating rate of 20 °C/min under N_2_ atmosphere.

The pore characteristics of the samples were studied by determining the nitrogen adsorption using a surface area and pore size analyzer (TriStarII 3020, Micromeritics Instruments Corp, Norcross, GA, USA) at −196 °C. MSNs were degassed at 350 °C for 12 h prior to analysis, while the drug-loaded samples were degassed at 120 °C for 12 h. The pore characteristics were determined according to the BET and BJH procedures from the adsorption branches of the isotherms.

The morphology and particle size of the prepared samples were characterized using a SEM (SU8000, Hitachi Ltd., Tokyo, Japan, operated at 10 kV). The samples were gold-plated prior to imaging. The morphology of the samples was also observed by TEM (JEM-200CX; Jeol Ltd., Tokyo, Japan).

### 3.3. Synthesis of Mesoporous Silica Nanoparticles

MSNs were synthesized under basic conditions using CTAB as the structure-directing agent, and TEOS as the silica source as reported by Radu with a little modification [37]. Briefly, CTAB (1.0 g, 2.75 mmol) was dissolved in a mixture of 480 mL of deionized water, and then 3.5 mL of 2.0 M sodium hydroxide was introduced into the CTAB solution at room temperature under constant stirring. The mixture was heated to 80 °C in an oil bath, then 5.0 mL of TEOS was added dropwise at a rate of 1 mL/min. The solution was stirred vigorously for 2 h at 80 °C. White precipitate that formed during the process was washed three times with ethanol and deionized water and freeze-dried under vacuum. The as-synthesized white powder was then calcined at 550 °C for 5 h to completely remove the surfactant.

### 3.4. Loading of Pyraclostrobin into HTCC-Capped MSNs

A typical procedure for the loading of pyraclostrobin into HTCC-capped MSNs was as follows: 60 mg of bare MSNs were dispersed in the pyraclostrobin-methanol solution (60 mg/mL, 1.0 mL). The suspension was sonicated at low power for 30 min. Afterwards, 2.4 mL of HTCC aqueous solution (25 mg/mL) was added dropwise to the suspension under sonification. After further sonification within 10 min, the pyraclostrobin-loaded HTCC-capped MSNs were collected by centrifugation at 10,000 rpm for 10 min, washed three times with deionized water, and were then freeze dried. The pyraclostrobin-loaded HTCC-capped MSNs were denoted as Py@MSNs-HTCC.

For pyraclostrobin-loaded bare MSNs, the resulting suspensions before the addition of HTCC aqueous solution were centrifuged, washed three times with deionized water, and were then freeze-dried. The pyraclostrobin-loaded bare MSNs were denoted as Py@MSNs.

### 3.5. Pyraclostrobin Loading Efficiency

The pyraclostrobiin LC of the nanoparticles was measured using high performance liquid chromatography (HPLC, 1200-DAD (Diode Array Detector), Agilent, Santa Clara, CA, USA). Generally, 10.0 mg of pesticide-loaded nanoparticles were dissolved in 25.0 mL of methanol under vigorous vortexing, and the clear solution was obtained for HPLC analysis. This procedure was repeated until the concentration of pyraclostrobin in the supernatant below the detection limit. The combined methanol solution was used for LC determination. The HPLC operating parameters were as follows: Venusil XBP-C18 column (Bonna-Agela Technologies Inc., Tianjin, China) (2.5 mm × 4.6 mm, 5 μm thickness); column temperature: 30 °C; mobile phase: (methanol: distilled water = 80:20); flow rate: 1.0 mL/min; DAD signals: 275 nm. The LC (%) of pyraclostrobin was calculated as follows: LC (%) = (weight of pesticide in nanoparticles/weight of nanoparticles) × 100%.

### 3.6. Pyraclostrobin Release

The weighed pyraclostrobin-loaded nanoparticles were dispersed in 200 mL of 30% methanol aqueous solution with 0.5% Tween-80 emulsifier, which was used as the release medium, in a D-800LS dissolution tester (Tianjin University, Tianjin, China) at a stirring speed of 100 rpm. The cumulative release rate of pyraclostrobin from the nanoparticles was calculated by measuring the concentrations of pyraclostrobin dissolved in the mixture solution at different times to evaluate the sustained release property. To measure the concentration, 1.0 mL of mixture was withdrawn at a given time intervals for HPLC analysis, followed by supplying the same volume of fresh buffer solution to ensure the same total solution volume. The accumulative pyraclostrobin release was calculated according to the following equation:

$$E_{r}=\frac{V_{e}\sum_{1}^{n-1}C_{i}+V_{0}C_{n}}{m_{pesticide}}\times 100\%$$

where *E_r_* is the accumulative release (%) of pyraclostrobin with respect to loaded pesticide; *V_e_* is the the sampled volume taken at a predetermined time interval (*V_e_* = 1.0 mL); *C_i_* is the pyraclostrobin concentration in release fluid at time *i*; *V*_0_ is the volume of release solution (200 mL); and *n* is the number of the samples. *m_pesticide_* is the total amount of pesticide enwrapped in the particles. The determination was repeated three times.

### 3.7. Bioactivity Studies of Pyraclostrobin-Loaded HTCC-Capped MSNs

In this experiment, the fungicidal activity of pyraclostrobin-loaded nanoparticles against *P*. *asparagi* was determined by the growth rate method. Mycelial discs (7 mm in diameter) of *P*. *asparagi* grown on potato dextrose agar (PDA) plates were cut from the margins of the colony and placed on PDA plates containing different concentrations of pyraclostrobin technical, pyraclostrobin-loaded nanoparticles and blank carrier MSNs-HTCC. Stock solutions of each sample were diluted with sterile molten PDA to obtain the desired concentrations. After incubation at 25 °C for one, two, three, four, five and six days, mycelial radial growth was measured and activity was expressed as the percentage of inhibition. Percentage of inhibition (%) was calculated as equal to the (colony diameter of control − colony diameter of treatment)/(colony diameter of control − mycelial discs diameter) × 100%.

## 4. Conclusions

In this paper, HTCC-coated mesoporous silica nanoparticles (MSNs) were fabricated successfully as a novel delivery system for pesticide. Electrostatic interaction and hydrogen bonding were considered to be the driving forces facilitating the formation of nanoparticles. By coating the MSNs with HTCC, the surface morphology changed and the zeta potential increased, becoming highly positive. The fungicide pyraclostrobin was loaded by a simple immersion method into bare and HTCC-coated silica nanoparticles. HTCC-coated MSNs exhibited better performance than bare MSNs for guest molecule loading efficiency. The pyraclostrobin-loaded nanoparticles showed an initial burst, followed by subsequent sustained release behavior. HTCC-coated MSNs released pesticide faster than bare MSNs in the initial stage. By taking advantage of their good biocompatibility and large dwelling space for guest chemicals, HTCC-capped MSNs exhibited good performance in fungicidal activity against *P*. *asparagi*. Even under half doses of pyraclostrobin technical, Py@MSNs-HTCC demonstrated almost the same activity, which obviously reduced the pesticide amounts applied and enhanced the utilization efficiency. With the advancement of cheaper and probably greener MSN synthesis techniques, this nanocarrier is viable for commercial exploitation will come true.

## Figures and Tables

**Table 1:** The loading content (LC) and zeta potential of pyraclostrobin-loaded nanoparticles ^a^.

Entry	Pesticide (mg)	HTCC (mg)	LC (%)	Zeta potential (mV)
1	30	60	28.5	28.9
2	45	60	34.8	30.2
3	60	60	40.3	32.4
4	75	60	41.6	28.8
5	40	40	32.4	29.2
6	60	40	39.5	31.6
7	60	80	41.2	34.6
8	60	/	26.7	−17.6

^a^ MSN of 60 mg was used. HTCC: *N*-2-hydroxypropyl trimethyl ammonium chloride chitosan.

**Table 2:** Mesoporous structure characterization of nanoparticles.

Sample	*S*_BET_ (m^2^/g)	*V*_t_ (cm^3^/g)	*D*_BJH_ (nm)
MSNs	1138.73	1.28	3.73
Py@MSNs	543.45	0.59	3.48
Py@MSNs-HTCC	29.02	0.22	59.79 *
Py@MSNs-HTCC calcined	960.17	1.14	3.88

* The silica nanoparticles lost their mesoporous structure characteristics after HTCC coating. *S*_BET_: Brunauer–Emmett–Teller (BET) surface area; *V*_t_: total pore volume; *D*_BJH_: Barrett–Joyner–Halenda (BJH) pore diameter.

**Table 3:** Fungicidal activity against *P*. *asparagi* on the 6th day. (CK: Control check).

Sample	Concentration (mg/L)	Colony Diameter (cm)	Percentage of Inhibition (%)
Pyraclostrobin technical (PT)	CK	6.4	/
	5.0	1.1	92.98
	20.0	0.9	96.49
Py@MSNs-HTCC (NP)	CK	6.4	/
	2.5	1.4	87.72
	10.0	1.0	94.74
MSNs-HTCC (CB)	CK	6.4	/
	5.0	3.7	26.32
	20.0	4.9	47.37

## Supplementary Materials

The following are available online at http://www.mdpi.com/2079-4991/6/7/126/s1.

## Abbreviations

The following abbreviations are used in this manuscript:

HPLC	High-performance liquid chromatography
LC	Loading content
MSN	Mesoporous silica nanoparticle
HTCC	*N*-(2-Hydroxyl)propyl-3-trimethylammonium chitosan chloride
TEOS	Tetraethylorthosilicate
CTAB	Cetyltrimethylammonium bromide
BET	Brunauer–Emmett–Teller
BJH	Barrett–Joyner–Halenda
FTIR	Fourier transform infrared
SEM	Scanning electron microscopy
TEM	Transmission electron microscopy
TGA	Thermogravimetric analysis
Py@MSNs-HTCC	Pyraclostrobin-loaded HTCC-capped MSNs
Py@MSN	Pyraclostrobin-loaded MSNs
