# Supplementary material for: Quaternized Chitosan-Capped Mesoporous Silica Nanoparticles as Nanocarriers for Controlled Pesticide Release
Source: Nanomaterials (Basel). 2016 Jun 28;6(7):126. doi: 10.3390/nano6070126 (PMC5224598; doi:10.3390/nano6070126)
Supplement: Supplementary file 1 [file nanomaterials-06-00126-s001.pdf]

Supplementary Materials: Quaternized Chitosan-Capped Mesoporous Silica Nanoparticles as Nanocarriers for Controlled Pesticide Release

Lidong Cao, Huirong Zhang, Chong Cao, Jiakun Zhang, Fengmin Li and Qiliang Huang

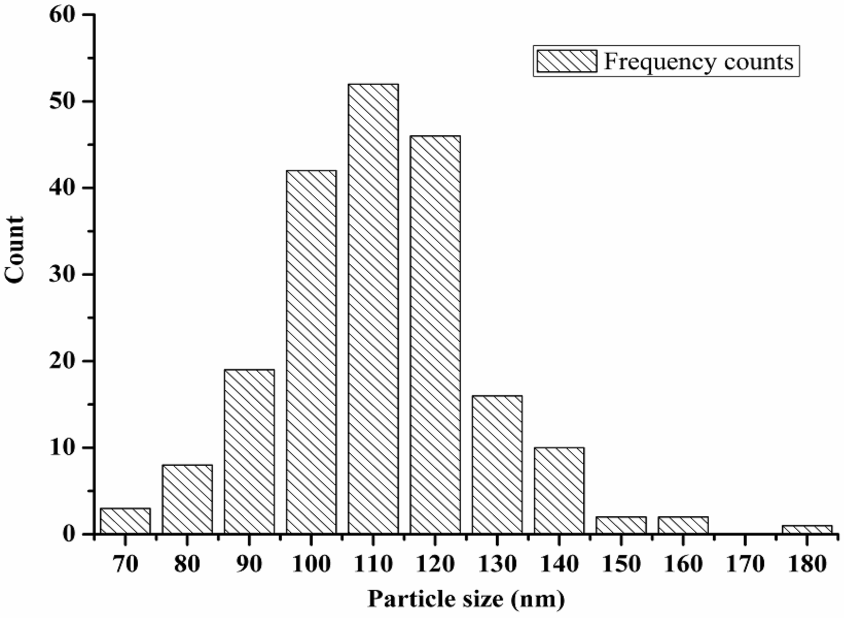

Figure S1. The distribution of particle size of Mesoporous silica nanoparticle (MSNs) based on the specimen numbers of 201.

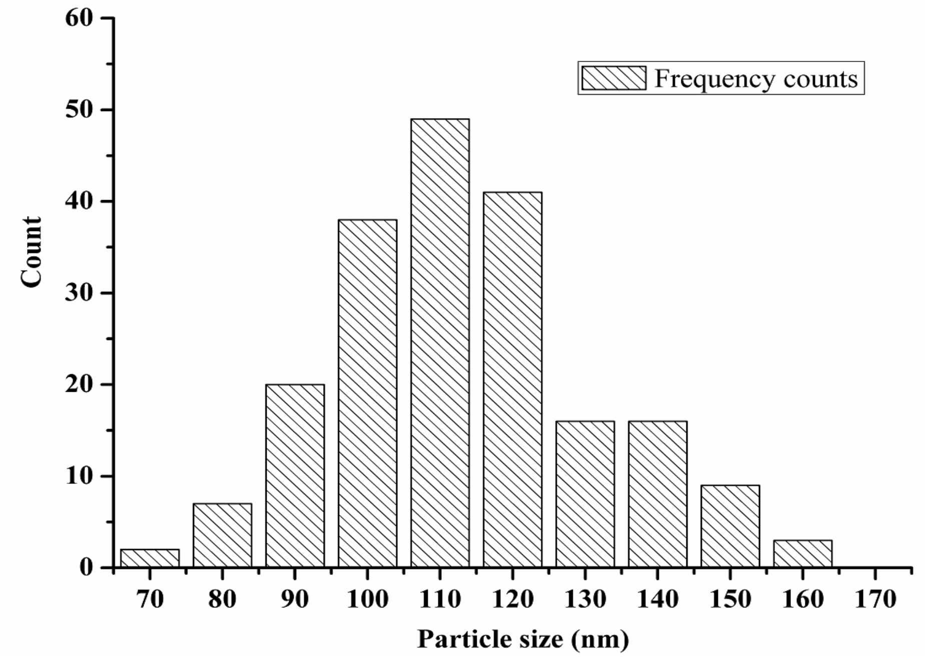

Figure S2. The distribution of particle size of pyraclostrobin-loaded HTCC-capped MSNs (Py@MSNs-HTCC) based on the specimen numbers of 201. HTCC: *N*-(2-Hydroxyl)propyl-3-trimethylammonium chitosan chloride.

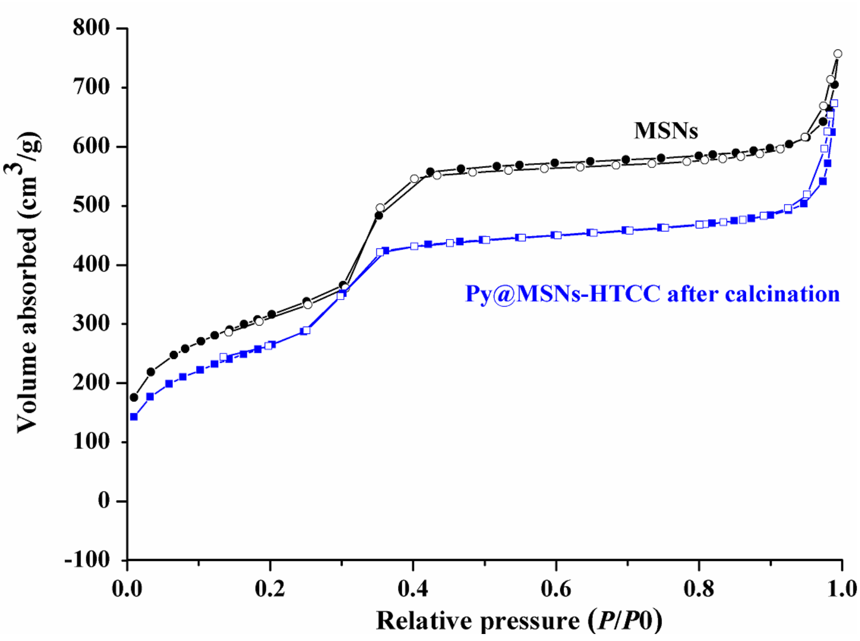

Figure S3. Nitrogen adsorption–desorption isotherms of MSNs and Py@MSNs-HTCC after calcination.

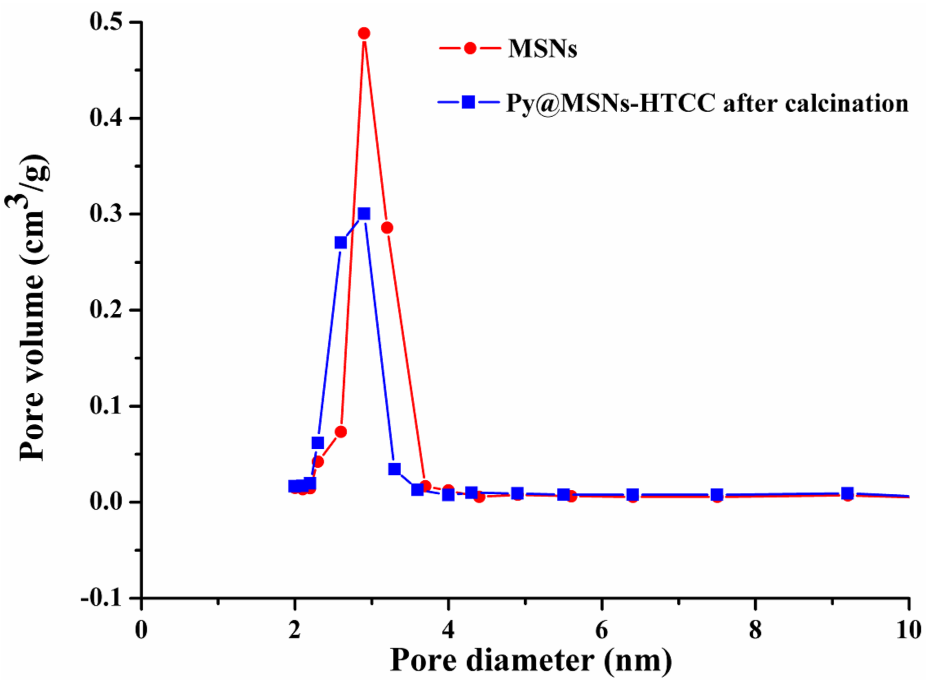

Figure S4. Barrett–Joyner–Halenda (BJH) pore-size-distribution curves of MSNs and Py@MSNs-HTCC after calcination.

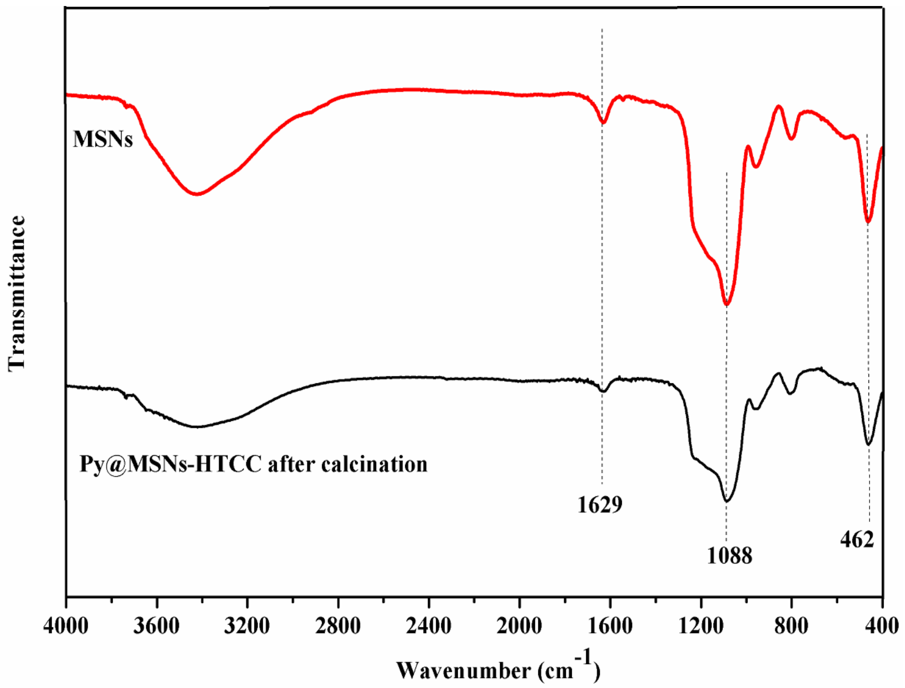

Figure S5. Fourier transform infrared (FTIR) of MSNs and Py@MSNs-HTCC after calcination.

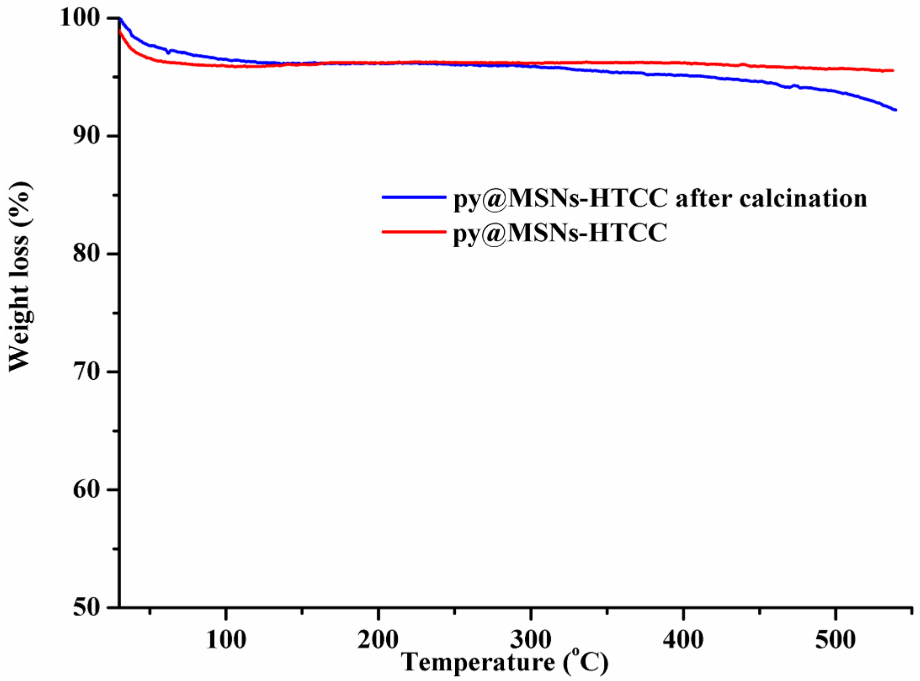

Figure S6. Thermogravimetric analysis (TGA) of MSNs and Py@MSNs-HTCC after calcination.

© 2016 by the authors. Submitted for possible open access publication under the terms and conditions of the Creative Commons Attribution (CC-BY) license (http://creativecommons.org/licenses/by/4.0/).
